# Effects of dual-task training on gross motor function, balance, and gait in children with cerebral palsy: a systematic review and meta-analysis

**DOI:** 10.3389/fneur.2026.1769434

**Published:** 2026-05-28

**Authors:** Bo Peng, Qi Sun

**Affiliations:** School of Rehabilitation Engineering, China Civil Affairs University, Beijing, China

**Keywords:** balance, cerebral palsy, dual-task training, GMFM, timed up and go

## Abstract

**Background:**

Children with cerebral palsy (CP) often experience cognitive–motor interference, which limits the ecological validity of traditional single-task rehabilitation. Dual-task training (DTT) integrates cognitive and motor tasks, potentially addressing this issue, but its effectiveness remains unclear due to protocol and outcome variability.

**Methods:**

A PRISMA 2020–compliant systematic review and meta-analysis of randomized controlled trials was conducted. Databases including PubMed, Web of Science, Embase, Scopus, the Cochrane Library, and CNKI were searched (2015 to October 16, 2025). Outcomes included balance capacity, gross motor function (GMFM Dimensions D/E), and functional mobility (Timed Up and Go, TUG). Risk of bias was assessed using RoB 2, and certainty of evidence with GRADE.

**Results:**

Eleven RCTs (*n* = 582) were included. Compared with conventional rehabilitation, DTT improved balance capacity (SMD = 1.11, 95% CI 0.89–1.33; *I*^2^ = 0%), GMFM Dimension D (MD = 5.05, 95% CI 1.30–8.79; *I*^2^ = 80%) and Dimension E (MD = 5.23, 95% CI 2.34–8.12; *I*^2^ = 41%), and TUG performance (MD = −2.49 s, 95% CI − 3.90 to −1.09; *I*^2^ = 69%). Overall risk of bias was “some concerns,” with certainty of evidence ranging from low to moderate. Egger’s test for balance capacity showed no small-study effects (*p* = 0.721).

**Conclusion:**

DTT may improve balance, gross motor function, and functional mobility in children with CP. Given outcome-specific heterogeneity and moderate certainty, results should be interpreted cautiously. Future well-powered trials with standardized protocols and longer follow-up are necessary.

**Systematic review registration:**

https://www.crd.york.ac.uk/PROSPERO/view/CRD42046832672, identifier (CRD42046832672).

## Introduction

Cerebral palsy (CP) is the most common cause of long-term physical disability in children, affecting approximately 1.6 per 1,000 live births globally (1, 2). Although CP is classically defined as a non-progressive disturbance of movement and posture due to early brain injury (3, 4), contemporary evidence indicates that its functional impact extends beyond motor impairment. Children with CP frequently present with deficits in cognition, attention, and executive functioning (5, 6), which can manifest as pronounced cognitive–motor interference during everyday activities. This heightened reliance on conscious attentional control may contribute to performance breakdown in complex or multitask environments.

Conventional rehabilitation is often dominated by repetitive single-task practice, which may have limited ecological validity and imperfect transfer to daily contexts that require divided attention (7–9). Motor learning research likewise suggests that skills acquired under minimally distracting conditions do not necessarily generalize to real-world situations characterized by competing attentional demands (10–12). Dual-task training (DTT) has therefore been proposed as an approach that explicitly trains motor skills under concurrent cognitive or secondary-task demands. Mechanistically, DTT has been hypothesized to reduce cognitive–motor interference and support more efficient control strategies, potentially facilitating greater motor automaticity and adaptive control through repeated practice under dual-task constraints (13–17). At the same time, most pediatric RCTs do not directly measure neural substrates, so these explanations should be interpreted as theory-informed rather than trial-confirmed. A recent systematic review and meta-analysis by Cortés-Pérez et al. synthesized randomized trials and reported overall benefits of DTT on postural balance and walking speed, while emphasizing substantial protocol variability and the need for cautious interpretation across outcomes (18). Earlier syntheses focusing on observational evidence primarily documented dual-task performance deficits but could not determine whether DTT reverses these deficits (19).

Despite the emerging evidence, clinically important uncertainties remain regarding the consistency of dual-task training effects across functional domains and across different dual-task modalities. In particular, gross motor function is frequently summarized using the total Gross Motor Function Measure score, yet domain-specific responsiveness may differ, especially for Dimension D and Dimension E, which are more directly related to standing and locomotor capacity. In addition, variability in dual-task content, training dose, and comparator characteristics is likely to contribute to heterogeneous effects and outcome-specific sensitivity. Building on the synthesis by Cortés-Pérez et al. (18), the present systematic review and meta-analysis was designed as an update and extension that incorporates additional randomized evidence, including trials published outside major English-language outlets, and evaluates outcomes using a prespecified domain framework. Accordingly, we aimed to synthesize randomized trials of dual-task training in children with CP with a focus on three clinically interpretable domains, gross motor function, balance capacity, and functional mobility, and to interpret pooled findings in the context of between-study heterogeneity to inform both rehabilitation decision-making and priorities for future trial design.

## Materials and methods

### Study design and registration

This systematic review and meta-analysis was conducted in strict adherence to the Preferred Reporting Items for Systematic Reviews and Meta-Analyses 2020 statement (20) and the methodological guidelines outlined in the Cochrane Handbook for Systematic Reviews of Interventions (21). The study protocol was prospectively registered with the International Prospective Register of Systematic Reviews under registration number CRD42046832672. To ensure methodological rigor, Methodological quality of this review was appraised using AMSTAR 2 (22).

### Data sources and search strategy

Studies were identified through systematic searches of PubMed, Scopus, Web of Science, the Cochrane Library, Embase, and China National Knowledge Infrastructure from January 2015 to 16 October 2025. Reference lists of included trials and relevant systematic reviews were also screened for additional eligible studies. The database searches were independently performed by two reviewers, Bo Peng and Qi Sun. All records were exported into a reference manager for de-duplication prior to screening.

Search strategies were developed using controlled vocabulary terms and free-text keywords. In PubMed, MeSH terms were combined with text words and searched in relevant fields. In Web of Science and Scopus, free-text terms were searched in titles, abstracts, and keywords using database-specific syntax. In Embase, controlled vocabulary and free-text terms were combined and mapped to appropriate fields. In CNKI, Chinese-language keywords were adapted to capture dual-task training concepts and pediatric cerebral palsy populations. Truncation, phrase searching, and Boolean operators were applied. The full, reproducible search strategy for PubMed and the adapted strategies for other databases are provided in Supplementary Table S1.

### Selection process

Records were de-duplicated before screening. Two reviewers, Bo Peng and Qi Sun, independently screened titles and abstracts, with inter-rater agreement quantified using Cohen’s kappa coefficient (23). Full texts were then assessed against prespecified eligibility criteria. Disagreements were resolved through discussion, and if consensus could not be reached, Lei Wu adjudicated eligibility decisions. Reference lists of included studies and relevant reviews were screened for additional eligible trials.

### Eligibility criteria

The inclusion criteria were prespecified according to the PICOS framework.

- Population (P): Children and adolescents aged 18 years or younger with a clinical diagnosis of cerebral palsy as defined by the trial authors. Trials with mixed-age samples were eligible only if pediatric data were reported separately or could be obtained. Gross Motor Function Classification System level was extracted when reported.
- Intervention (I): Dual-task training, defined as structured practice in which a primary motor activity was performed concurrently with a secondary cognitive task or a secondary motor task. Dual-task training could be delivered as a standalone intervention or as an add-on component to conventional rehabilitation.
- Comparator (C): Conventional rehabilitation, usual care, routine physical therapy, or other non-dual-task interventions. Comparator programs were required to represent active rehabilitation and to omit structured concurrent dual-task practice as an explicit therapeutic component. Core elements typically included strength or flexibility training, postural control or balance exercises, gait training, and task-oriented practice, with intervention content and dose extracted from each trial.
- Outcomes (O): Studies reporting at least one quantitative outcome within the prespecified domains of gross motor function, balance capacity, or functional mobility. Outcome eligibility was defined by domains rather than by specific instruments. When multiple measures were reported within a domain, a prespecified selection rule was applied to avoid double counting and to enhance comparability across studies.
- Study design (S): Randomized controlled trials. Pilot randomized trials were eligible when randomization was reported and outcome data were extractable. Sensitivity analyses were prespecified to examine the influence of pilot trials on pooled estimates.

Studies were excluded if they:

- used non-randomized, quasi-experimental, or observational designs;
- enrolled participants with progressive neurological disorders or conditions likely to confound motor outcomes independent of cerebral palsy;
- did not report sufficient data for effect size calculation and the required data could not be derived from available statistics;
- were conference abstracts, reviews, meta-analyses, editorials, letters, or opinion pieces.

### Data extraction

Two reviewers, Bo Peng and Qi Sun, independently extracted data using a standardized form. Extracted information included publication details, participant characteristics, cerebral palsy subtype when reported, GMFCS level, intervention content and dose, comparator characteristics, outcomes, and assessment time points. Extracted data were cross-checked for accuracy. Disagreements were resolved by consensus, with Lei Wu adjudicating when needed. For trials with multiple eligible intervention arms, only the most relevant dual-task training arm was included per comparison to avoid double counting. When required data were missing or unclear, we attempted to derive values from available statistics or contact study authors.

### Outcome measures

Outcome eligibility was prespecified by outcome domains. The primary outcome domains were gross motor function, balance capacity, and functional mobility. Gross motor function was extracted from the Gross Motor Function Measure, including the total score and, when reported, Dimension D and Dimension E to reflect standing-related and locomotor capacity. Balance capacity was assessed using clinical balance scales, specifically the Pediatric Balance Scale and the Berg Balance Scale, which share an identical scoring range and direction and were pooled to represent overall balance capacity. Functional mobility was evaluated using time-based mobility tests, primarily the Timed Up and Go test, including modified versions when explicitly defined by the trial. When multiple measures within the same domain were reported, prespecified selection rules were applied to avoid double counting, and the instrument-to-domain mapping and selection rules are documented in Supplementary material.

### Risk of bias assessment

Two reviewers independently assessed methodological quality using the revised Cochrane Risk of Bias tool for randomized trials (RoB 2) (24). Risk of bias was assessed for each primary outcome. The following domains were evaluated: bias arising from the randomization process, bias due to deviations from intended interventions, bias due to missing outcome data, bias in measurement of the outcome, and bias in selection of the reported result. Each domain and the overall risk of bias were judged as low risk, some concerns, or high risk. Disagreements were resolved through discussion and re-examination of the original reports, with arbitration by a third reviewer when required.

### Certainty of evidence assessment

We assessed the certainty of evidence for each primary outcome domain using the Grading of Recommendations Assessment, Development and Evaluation approach (25). Evidence from randomized trials started at high certainty and was downgraded based on risk of bias, inconsistency, indirectness, imprecision, and publication bias. Inconsistency was evaluated considering both statistical heterogeneity and the plausibility of clinical or methodological explanations for between-study variability. Indirectness was judged based on the similarity of populations, interventions, comparators, and outcomes to the review question. Imprecision was judged using total sample size and confidence interval width for pooled estimates. Publication bias was considered using funnel plot patterns when sufficient studies were available and, when applicable, by small-study effect assessments. Summary of Findings tables were generated using GRADEpro GDT (McMaster University and Evidence Prime, 2024) to present pooled effects and certainty ratings.

### Statistical analysis

Meta-analysis was performed using Review Manager version 5.3 (26). For continuous outcomes, MD with 95% confidence intervals was used when outcomes were reported on an identical metric, including instruments with the same scoring range and direction. Accordingly, PBS and BBS were pooled using MD because both scales range from 0 to 56 and higher scores indicate better balance. SMD with 95% confidence intervals was used when outcomes within the same domain were measured using different scales or units. Outcome direction was harmonized so that positive values indicated improvement. Heterogeneity was assessed using the Chi-square test and the *I*^2^ statistic, and interpreted using conventional thresholds (27). Random-effects models were used when heterogeneity was substantial, otherwise fixed-effect models were applied. Robustness was examined using leave-one-out sensitivity analyses. Subgroup analyses were conducted by dual-task modality, intervention duration, and age, and were interpreted as exploratory due to limited studies per subgroup. Publication bias was assessed using funnel plots when sufficient studies were available. Egger’s regression test was applied in Stata (StataCorp LLC, College Station, TX, USA) when at least 10 studies were available for an outcome.

## Results

### Study selection

**Figure 1:**
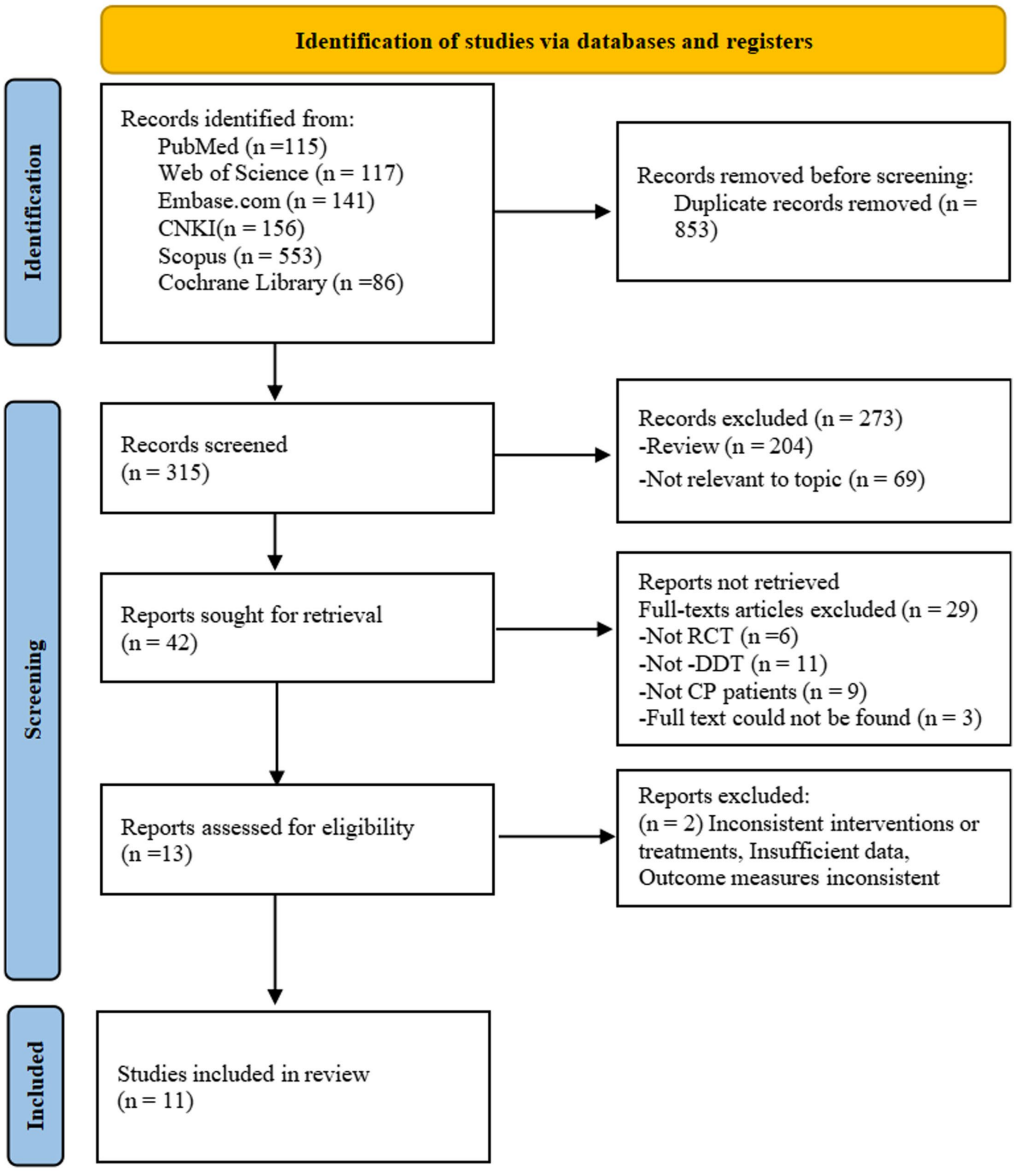
Flowchart of literature screening.

A total of 1,168 records were identified from PubMed (*n* = 115), Web of Science (*n* = 117), Embase (*n* = 141), CNKI (*n* = 156), Scopus (*n* = 553), and the Cochrane Library (*n* = 86). After removing 853 duplicates, 315 records were screened and 273 were excluded; 42 full texts were assessed and 31 were excluded due to non-randomized design (*n* = 6), intervention not meeting dual-task training criteria (*n* = 11), non-CP pediatric samples (*n* = 9), full text not retrievable (*n* = 3), or other reasons such as inconsistent intervention content and insufficient or non-comparable outcome data (*n* = 2), leaving 11 randomized controlled trials for qualitative synthesis and meta-analysis (Figure 1).

### Characteristics of included studies

A total of 11 randomized controlled trials were included, comprising 582 children with cerebral palsy, with 292 allocated to the experimental groups and 290 to the control groups (Table 1). The studies were published between 2020 and 2025 and were conducted across multiple regions, including Saudi Arabia, Pakistan, Lithuania, South Korea, China, Egypt, Canada, and Turkey. Interventions were classified *a priori* as motor-cognitive dual-task training or motor-motor dual-task training. Motor-cognitive dual-task training was applied in most trials (13–15, 28–33), where participants performed concurrent cognitive tasks such as arithmetic, verbal fluency, memory recall, or game-based interactions while undertaking balance or gait activities. Motor-motor dual-task training was reported in (15, 34, 35), and typically combined a secondary motor task such as object carrying or ball handling with the primary motor activity. Outcome measures were reorganized into three prespecified domains to improve comparability across trials. Balance capacity was assessed using clinical balance scales, namely the Pediatric Balance Scale and the Berg Balance Scale (13–15, 28–35). These scales share an identical scoring range and direction, and were treated as measures of the same construct for synthesis when data were available and eligible for quantitative pooling. Gross motor function was evaluated using the Gross Motor Function Measure, with extraction of the total score and, when available, Dimension D and Dimension E to reflect standing-related and locomotor capacity (15, 28, 31, 32, 34). Functional mobility was assessed using time-based mobility tests, primarily the Timed Up and Go test, including modified versions when defined by the original trials (13, 15, 29, 31, 32).

**Table 1:** Summary of basic characteristics of included studies.

Study (country)	Participants (*n*; age; sex; GMFCS)	Intervention comparisons	Intervention details (protocol and dosage)	Outcome measures
Abuzaid et al. 2024 (32) (Saudi Arabia)	E: *n* = 20; Age 9.3 ± 1.4; M/F: 9/11; GMFCS I–II	E: Motor-cognitive DTT	E: Walking + Cognitive tasks (naming animals). 30 min/session, 2×/week, 8 weeks.	GMFM (D, E), PBS, TUG
	C: *n* = 20; Age 9.3 ± 1.4; M/F: 12/8; GMFCS I–II	C: Standard Rehab	C: Standard rehabilitation program.	
Kamran et al. (51) (Pakistan)	E: *n* = 26; Age 8.6 ± 1.9; M/F: 15/11; GMFCS II–III	E: Motor-cognitive DTT	E: Treadmill walking + Cognitive tasks (5 distinct tasks). 15 min DTT/session, 8 weeks.	PBS, GMFM
	C: *n* = 26; Age 8.5 ± 2.0; M/F: 12/14; GMFCS II–III	C: Conventional Therapy	C: Balance and gait exercises.	
Kvedaravičienė et al. 2020 (34) (Lithuania)	E: *n* = 10; Age 10.4 ± 1.2; M/F: NR; GMFCS I–II	E: Motor-motor DTT + Routine PT	E: Unstable surface walking + Motor tasks (ball throwing). 20 min DTT + 20 min PT, 5×/week, 3 weeks.	PBS, GMFM (D, E)
	C: *n* = 10; Age 10.4 ± 1.2; M/F: NR; GMFCS I–II	C: Routine PT	C: 40 min Routine PT.	
Lee et al. 2021 (29) (South Korea)	E: *n* = 7; Age 9.4 ± 2.1; M/F: 4/3; GMFCS I–III	E: Motor-cognitive DTT	E: Balance on unstable surface + Cognitive tasks. 30 min/session, 2×/week, 8 weeks.	Static/Dynamic Balance, GMFM (D, E)
	C: *n* = 7; Age 9.4 ± 2.3; M/F: 3/4; GMFCS I–III	C: NDT	C: Neurodevelopmental treatment (NDT).	
Luo G et al. 2022 (31) (China)	E: *n* = 18; Age 4.5 ± 0.4; M/F: 12/6; GMFCS I–II	E: Motor-cognitive DTT	E: Treadmill walking + Cognitive tasks. 50 min/session, 5×/week, 4 weeks.	PBS, TUG, GMFM (D, E)
	C: *n* = 16; Age 4.6 ± 0.5; M/F: 10/6; GMFCS I–II	C: Single-task Training	C: Treadmill training alone.	
Luo S et al. 2021 (33) (China)	E: *n* = 29; Age 7.6 ± 1.4; M/F: 17/12; GMFCS I–II	E: DTT + Routine PT	E: 20 min DTT + 25 min NDT. 5×/week, 8 weeks.	BBS, Gait parameters
	C: *n* = 29; Age 7.5 ± 1.4; M/F: 15/14; GMFCS I–II	C: Routine PT	C: NDT, OT, Speech therapy, Sensory integration, FES.	
Mahmoud et al. 2023 (35) (Egypt)	E: *n* = 17; Age 7.7 ± 2.2; M/F: 10/7; GMFCS I–II	E: Motor-motor DTT	E: Balance board + Throwing/catching. 30 min/session (+ Routine PT), 3×/week, 8 weeks.	PBS
	C: *n* = 17; Age 7.6 ± 1.7; M/F: 10/7; GMFCS I–II	C: Vestibular Training	C: Vestibular training + Routine PT.	
Szturm et al. 2022 (14) (Canada)	E: *n* = 10; Age 6.3 ± 2.3; M/F: 7/3; GMFCS I–III	E: Motor-cognitive DTT	E: Balance exercises + Computer games. 45 min/session, 3×/week, 12 weeks.	PBS
	C: *n* = 10; Age 6.3 ± 2.3; M/F: 7/3; GMFCS I–III	C: Conventional Therapy	C: Conventional balance exercises.	
Uysal et al. 2024 (13) (Turkey)	E: *n* = 15; Age 9.8 ± 2.6; M/F: 10/5; GMFCS I–II	E: Motor-cognitive DTT	E: Balance/Walking + Cognitive tasks (math/attention). 30 min/session, 3×/week, 12 weeks.	PBS, TUG, Static Balance
	C: *n* = 15; Age 9.7 ± 2.8; M/F: 10/5; GMFCS I–II	C: Conventional Therapy	C: Conventional lower limb exercises.	
Yang et al. 2023 (28) (China)	E: *n* = 40; Age 8.9 ± 1.5; M/F: 24/16; GMFCS I–II	E: DTT + Family Rehab	E: Motor-cognitive DTT. 20 min/session, 3 months.	GMFM (D, E), BBS,
	C: *n* = 40; Age 8.9 ± 1.5; M/F: 24/16; GMFCS I–II	C: Routine rehabilitation training	C: Routine rehabilitation training.	
Zhang et al. 2023 (30) (China)	E: *n* = 100; Age 3.2 ± 0.7; M/F: 58/42; GMFCS I–II	E: DTT + Physical Rehab	E: Walking + Cognitive tasks. 20–30 min/session, 5×/week, 3 months.	GMFM-88, BBS
	C: *n* = 100; Age 3.1 ± 0.6; M/F: 61/39; GMFCS I–II	C: Routine PT	C: Paraffin, NDT, Sensory integration, FES.	

BBS, Berg Balance Scale; C, Control Group; DTT, Dual-Task Training; E, Experimental Group; FES, Functional Electrical Stimulation; GMFCS, Gross Motor Function Classification System; GMFM, Gross Motor Function Measure; M/F, Male/Female; NDT, Neurodevelopmental Treatment; NR, Not Reported; OT, Occupational Therapy; PBS, Pediatric Balance Scale; PT, Physical Therapy; TUG, Timed Up and Go Test.

### Risk of bias

**Figure 2:**
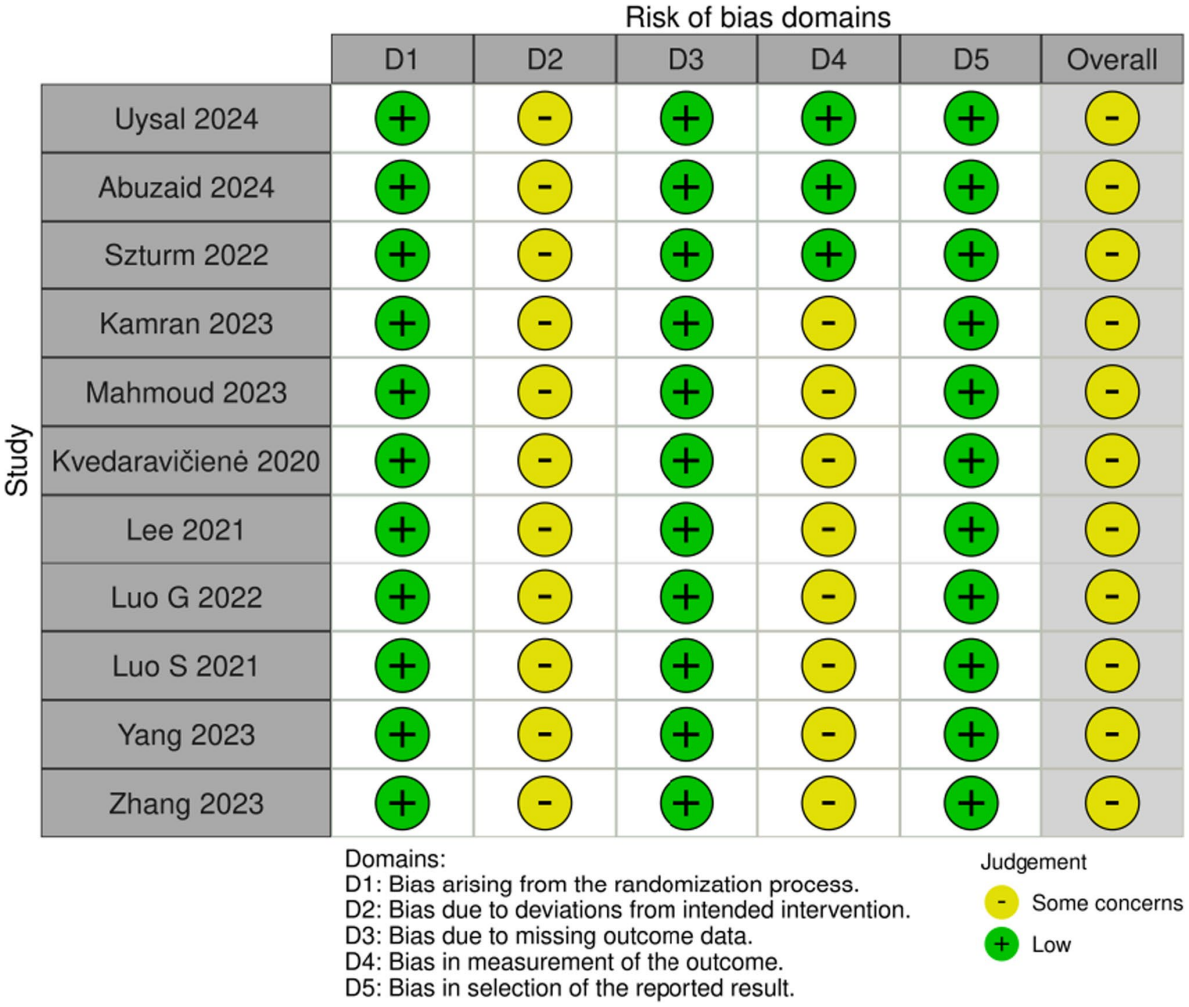
Risk of bias assessment of included studies using the RoB 2 tool.

Risk of bias was assessed using the revised Cochrane Risk of Bias tool for randomized trials (RoB 2) and is summarized in Figure 2. Across the 11 included trials, all studies were judged at low risk of bias for Domain 1 (randomization process), Domain 3 (missing outcome data), and Domain 5 (selection of the reported result). In contrast, all trials were rated as some concerns for Domain 2 (deviations from intended interventions). For Domain 4 (measurement of the outcome), three trials were judged at low risk (13, 14, 32), whereas the remaining studies were rated as some concerns (15, 28–31, 33–35). Consequently, the overall risk of bias was rated as some concerns for all included studies, primarily driven by deviations from intended interventions and, in some trials, by outcome measurement considerations.

### Meta-analysis results

#### Gross motor function

**Figure 3:**
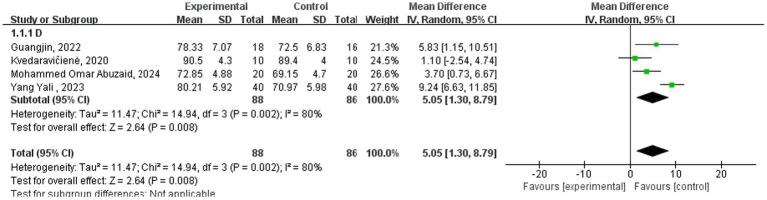
GMFM Dimension D forest plot.

GMFM Dimension D analysis is shown in Figure 3. Analysis of four studies (*n* = 174) revealed a significant benefit of DTT for GMFM Dimension D (MD = 5.05, 95% CI [1.30, 8.79], *p* = 0.008), despite high heterogeneity (*I*^2^ = 80%, *χ*^2^ = 14.94, df = 3, *p* = 0.002). The overall pooled effect indicates that DTT significantly improves GMFM Dimension D (*Z* = 2.64, *p* = 0.008).

**Figure 4:**
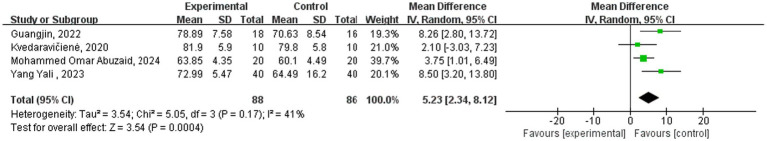
GMFM Dimension E forest plot.

Analyses for GMFM Dimension E are presented in Figure 4. Analysis of four studies (*n* = 174) revealed a significant benefit of DTT for GMFM Dimension E (MD = 5.23, 95% CI [2.34, 8.12], *p* = 0.0004), with moderate heterogeneity (*I*^2^ = 41%, *χ*^2^ = 5.05, df = 3, *p* = 0.17). The overall pooled effect indicates that DTT significantly improves GMFM Dimension E (*Z* = 3.54, *p* = 0.0004).

### Balance capacity

**Figure 5:**
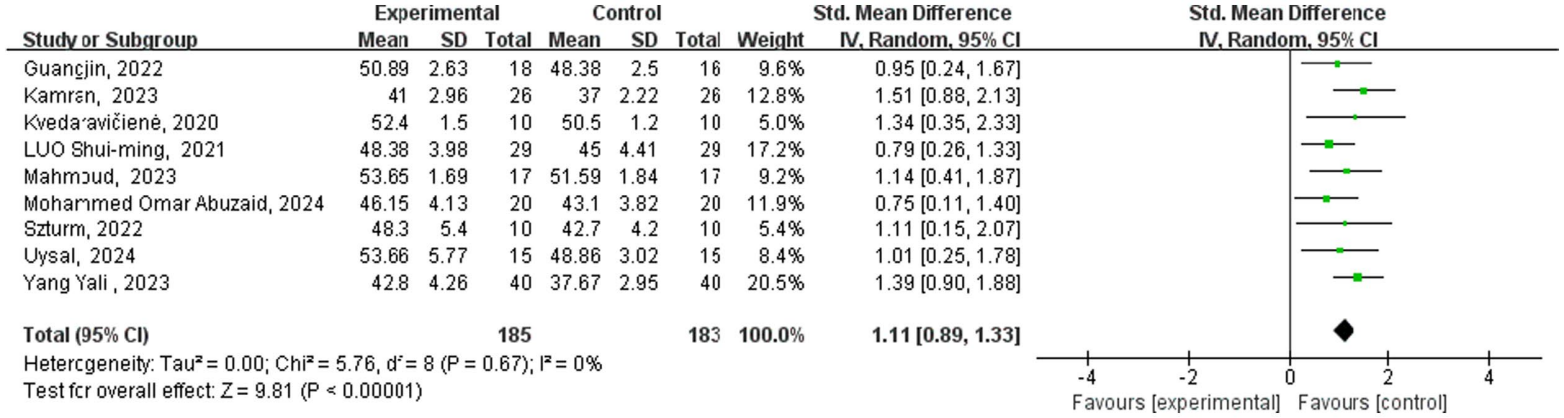
Balance function forest plot.

Data from nine comparisons (*n* = 368) demonstrated that DTT resulted in significantly greater improvements in balance capacity compared to controls (SMD = 1.11, 95% CI [0.89, 1.33], *p* < 0.00001), as shown in Figure 5. Heterogeneity was low (*I*^2^ = 0%, *χ*^2^ = 5.76, df = 8, *p* = 0.67).

### Functional mobility

**Figure 6:**
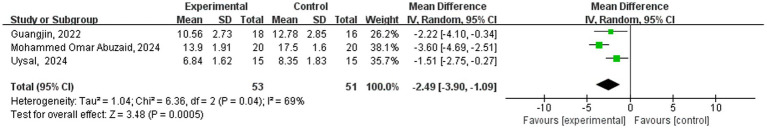
Timed up and go forest plot.

Data from three studies (*n* = 104) revealed a significant improvement in functional mobility as measured by the Timed Up and Go (TUG) test following DTT (MD = −2.49, 95% CI [−3.90, −1.09], *p* = 0.0005), as shown in Figure 6. Moderate heterogeneity was observed (*I*^2^ = 69%, *χ*^2^ = 6.36, df = 2, *p* = 0.04).

### Sensitivity analysis and investigation of heterogeneity

**Figure 7:**
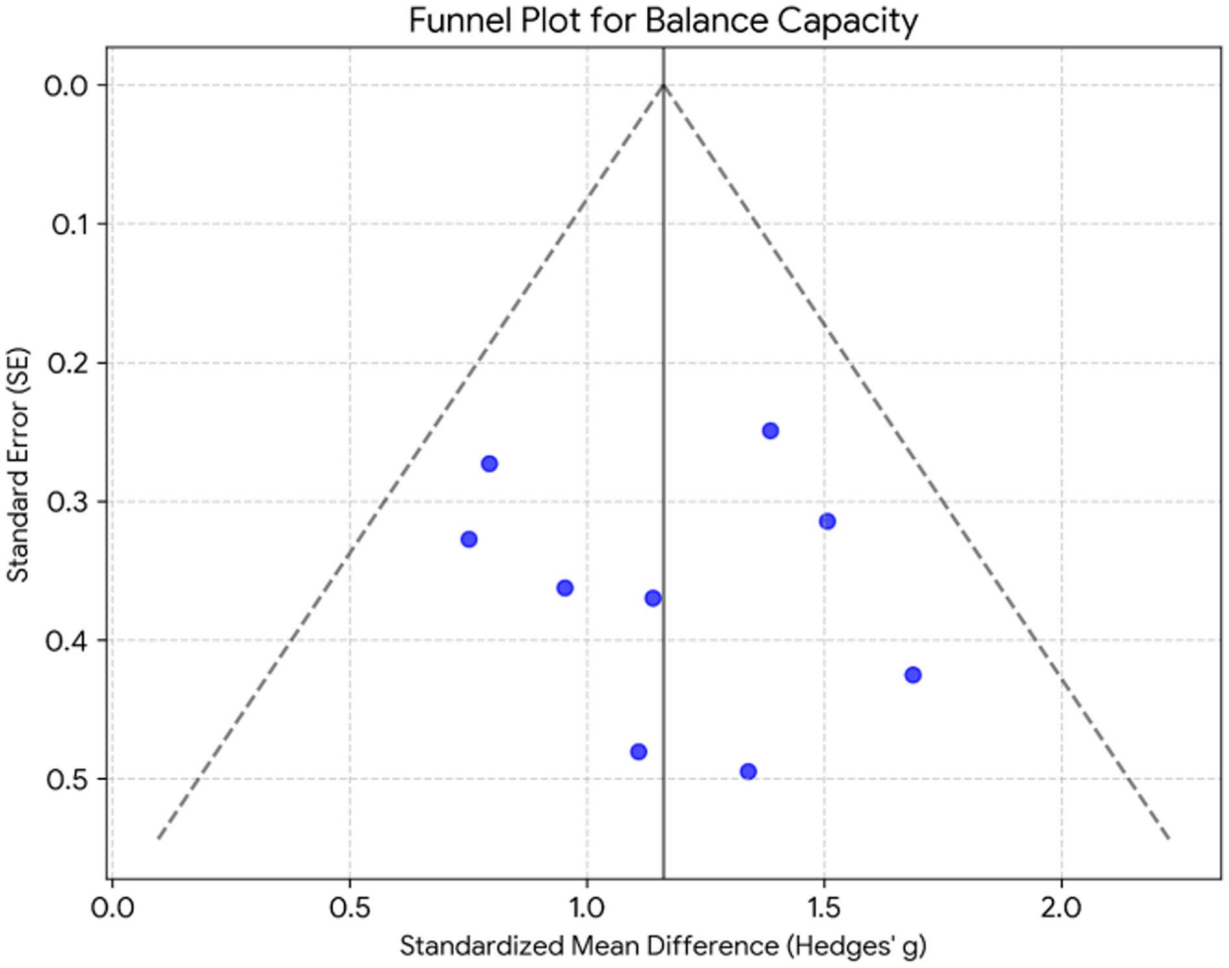
Funnel plot.

Leave-one-out sensitivity analyses were performed for all outcomes to assess the robustness of the results. For balance capacity, there was no statistical heterogeneity (*I*^2^ = 0%), and thus, subgroup analyses or meta-regression were not deemed necessary. The pooled effect remained robust and highly significant after the removal of any single study, confirming the consistency of the dual-task training effect. For outcomes exhibiting moderate-to-high heterogeneity (GMFM Dimensions D and E, and TUG), the observed heterogeneity could not be fully resolved through sensitivity analyses. Given the limited number of available studies (*k* ≤ 4), pre-planned subgroup analyses or meta-regression were not conducted, as these would have been severely underpowered and prone to spurious conclusions. As such, the results for these outcomes should be interpreted with caution. Nonetheless, the overall direction of the effect remained consistent, indicating that the positive findings were not driven by any single outlier (Figure 7).

### Publication bias

Publication bias was quantitatively assessed using Egger’s test for the primary outcome with the largest number of included studies (Balance Capacity). Visual inspection of the funnel plot demonstrated a generally symmetrical distribution of effect sizes around the pooled estimate. This was statistically confirmed by Egger’s linear regression test, which yielded an intercept of 0.58 (*p* = 0.721). The non-significant result (*p* > 0.05) indicates no evidence of small-study effects or publication bias, further supporting the robustness and validity of the meta-analysis results.

### Certainty of evidence (GRADE assessment)

The certainty of the evidence for the main outcomes is summarized in Table 2. Gross Motor Function: The evidence for improvements in GMFM Dimensions D and E was rated as low. The certainty was downgraded due to serious inconsistency (*I*^2^ = 41–80%), reflecting variability in intervention protocols, and imprecision resulting from the relatively small pooled sample size (*n* = 174). Balance Capacity: The certainty of the evidence for balance capacity (pooled PBS and BBS) was rated as moderate. The data showed zero statistical heterogeneity (*I*^2^ = 0%) and precise confidence intervals. The rating was downgraded by only one level due to the inherent risk of bias in the included trials, specifically the lack of blinding for participants and therapists. Functional Mobility: The evidence for functional mobility, measured by the TUG test, was also rated as low. The downgrading factors included serious inconsistency (*I*^2^ = 69%) and imprecision due to the limited number of participants (*n* = 104) across the three included trials.

**Table 2:** Dual-task training compared to conventional rehabilitation for children with cerebral palsy.

Outcomes	No of participants (studies)	Relative effect (95% CI)	Certainty of the evidence (GRADE)	Comments
Gross motor function (GMFM total score) assessed with: GMFM-88/66	174 (4 RCTs)	MD 5.23 higher (2.34–8.12)	⨁⨁◯◯ LOW^b,c^	Significant improvement. Downgraded due to serious inconsistency (*I*^2^ = 41–80%) and imprecision (small sample size).
Balance capacity assessed with: PBS and BBS	368 (9 RCTs)	SMD 1.11 higher (0.89–1.33)	⨁⨁⨁◯ MODERATE^a,c^	Highly significant improvement. Heterogeneity was exceptionally low (*I*^2^ = 0%). Downgraded one level due to risk of bias (lack of blinding).
Functional mobility assessed with: timed up and go	104 (3 RCTs)	MD 2.49 s lower (3.9–1.09)	⨁⨁◯◯ LOW^b,c^	Significant improvement. TUG time was reduced. Downgraded due to serious inconsistency (*I*^2^ = 69%) and imprecision (small sample size).

^a^Downgraded one level for risk of bias due to lack of blinding for participants and therapists, which is inherent in exercise interventions. ^b^Downgraded one level for inconsistency due to moderate-to-high heterogeneity (*I*^2^ > 50%), likely caused by variations in task complexity. ^c^Downgraded one level for imprecision as the total sample size is small (*N* < 400).

## Discussion

This systematic review and meta-analysis aggregated data from 11 RCTs involving 582 children with CP, providing updated evidence on the use of DTT in pediatric rehabilitation. Compared with conventional physical therapy, DTT was associated with improvements in balance capacity, standing and locomotor gross motor function (GMFM Dimensions D and E), and functional mobility (Timed Up and Go). Given that the certainty of evidence ranged from low to moderate, these findings support DTT as a promising approach, while the estimated effects should be interpreted cautiously in light of protocol diversity and outcome-specific heterogeneity across trials. The following sections interpret these outcome patterns in relation to protocol heterogeneity, measurement characteristics, and the broader pediatric rehabilitation literature.

### Outcome interpretation and measurement considerations

Balance capacity showed statistically consistent effects across trials, suggesting it may represent a relatively robust responder domain within the current evidence base. Notably, the Pediatric Balance Scale and the Berg Balance Scale incorporate task-oriented postural control demands (e.g., transitions, turning, reaching) that align closely with the cognitive motor integration targeted in DTT, and both scales have been reported to demonstrate good reliability and responsiveness in children with CP (36, 37). Nevertheless, low statistical heterogeneity should not be interpreted as the absence of clinical variability, as included trials differed in DTT content and dose as well as the nature of comparator interventions. Consistent with the GRADE rating (moderate certainty), the balance findings support clinical promise, while the magnitude of benefit should still be interpreted with awareness of methodological limitations, particularly performance bias inherent to exercise-based trials.

The contrasting heterogeneity profile high for Dimension D but moderate for Dimension E suggests that the magnitude of GMFM gains may be sensitive to protocol-level factors, including the type of secondary task (motor cognitive vs. motor motor), training dose (15–50 min/session), and the variability of comparator interventions. Within the ICF framework, rehabilitation outcomes can be distinguished between capacity and performance (38). In this context, the GMFM is commonly used as a capacity-oriented measure, and its responsiveness may be constrained in higher-functioning children because of ceiling effects and limited sensitivity to movement quality or automaticity (39). In higher-functioning children (e.g., GMFCS I–II), ceiling effects and limited sensitivity to changes in movement quality or automaticity may constrain its responsiveness (40, 41), which may partially explain why effect sizes vary across studies even when the overall direction favors DTT. Accordingly, the low certainty of evidence GRADE for GMFM outcomes indicates that DTT may improve standing and locomotor capacity, but the precision and generalizability of the estimated effects remain limited and require confirmation in larger, better-standardized trials.

The TUG reflects transitional mobility and requires dynamic task organization, which may be more aligned with real-world “performance” demands than capacity-based measures alone (17, 19, 42, 43). However, the small number of contributing trials and the inconsistency across protocols limit firm inference regarding effect magnitude and sources of variability. Therefore, consistent with the low GRADE certainty for TUG, current evidence supports a potential benefit of DTT on functional mobility, while emphasizing that standardized progression of dual-task difficulty and better-defined, dose-matched comparators are needed to clarify whether the observed improvement is stable across populations and settings.

### Potential neural mechanisms underlying functional improvements

The functional improvements observed in this review may be partly explained by DTT’s potential to reduce cognitive–motor interference and support adaptive motor learning. Evidence from dual-task walking research indicates that concurrent task performance can increase reliance on executive-control resources and alter cortical engagement patterns, depending on the type of interference (44). In children with CP, dual-task conditions have been shown to challenge gait and postural performance, consistent with heightened cognitive motor interference (17, 19, 45). In this context, repeated cognitive motor integration practice may reduce the need for continuous conscious monitoring and promote more efficient task coordination. Importantly, while explicit learning mechanisms may be impaired in some children with CP, particularly those with hemiplegia, a preserved capacity for implicit learning is often reported (45, 46), which may support practice related gains.

Indirect support for improved efficiency also comes from neuroimaging and biological studies showing training-related reductions in activation of executive control regions (47) and changes in plasticity related markers (48). In addition, when aerobic components are embedded in DTT protocols, executive control processes such as inhibitory function may be facilitated, which could be relevant to dual-task performance (32). However, these mechanistic explanations remain hypothesis-generating because most included RCTs did not directly assess neural or molecular correlates; future studies incorporating objective mechanistic measures (e.g., fNIRS) are needed to test these pathways (49, 50).

### Limitations and future directions

Despite adherence to PRISMA 2020 standards and the use of the RoB 2 tool, several limitations should be acknowledged. First, heterogeneity was outcome-specific, with more consistent effects for balance capacity but greater variability for gross motor function and functional mobility. This likely reflects differences in dual-task paradigms, intervention dose and progression, and the content and intensity of comparator interventions, which were not fully standardized across trials. Second, although the overall sample size was moderate, the number of trials contributing to some outcomes was limited. As a result, subgroup analyses and meta-regression were underpowered, preventing quantitative evaluation of potential effect modifiers (e.g., task type, dosage, GMFCS level, age, CP subtype). Third, the certainty of evidence ranged from low to moderate, driven by common risk-of-bias concerns, outcome inconsistency, and imprecision due to small samples. Fourth, most evidence focused on motor–cognitive dual-task training, with relatively limited data for motor–motor paradigms, restricting comparative inference across DTT modalities. Finally, most trials reported only immediate post-intervention outcomes, with scarce long-term follow-up, limiting conclusions regarding retention and real-world transfer of functional gains.

Future studies should prioritize protocol standardization and transparent reporting to enhance comparability and enable cumulative evidence synthesis. Specifically, researchers should (i) define and titrate dual-task difficulty and report progression rules, (ii) employ dose-matched and well-characterized comparators to isolate the incremental effect of dual-task components, and (iii) stratify randomization and prespecify analyses by GMFCS level, age, and CP subtype to identify responder profiles. Adoption of a core outcome set for dual-task interventions would harmonize outcome selection and assessment timing, and longer follow-up periods are needed to evaluate retention and maintenance. Incorporating objective mechanistic measures (e.g., fNIRS) may help test hypothesized pathways.

Given the potential for performance bias in exercise-based interventions and ethical constraints in randomized trials involving children with chronic neurological conditions, future studies should also consider assessor blinding, standardized intervention delivery, objective adherence monitoring, and ethically feasible designs (e.g., crossover, stepped-wedge, or observational studies). These strategies can help minimize bias, enhance methodological rigor, and ensure that findings are both scientifically robust and ethically responsible.

## Conclusion

Current evidence suggests that dual-task training may improve balance capacity, standing/locomotor gross motor function (GMFM Dimensions D and E), and functional mobility (Timed Up and Go) in children with cerebral palsy compared with conventional rehabilitation. Effects on balance were consistent, whereas GMFM and TUG effects varied across studies, reflecting outcome- and protocol-dependent responsiveness. Given the low-to-moderate certainty of evidence and limited ability to examine effect modifiers, these findings should be interpreted cautiously. Further well-designed trials that implement standardized protocols, bias control measures, and ethically feasible designs are warranted to confirm generalizability, durability, and mechanistic underpinnings of dual-task interventions in this population.

## Data Availability

The original contributions presented in the study are included in the article/Supplementary material, further inquiries can be directed to the corresponding author.
