# Supplementary material for: Effects of dual-task training on gross motor function, balance, and gait in children with cerebral palsy: a systematic review and meta-analysis
Source: Front Neurol. 2026 May 28;17:1769434. doi: 10.3389/fneur.2026.1769434 (PMC13253374; doi:10.3389/fneur.2026.1769434)
Supplement: Supplementary file 1 [file Data_Sheet_1.zip › Supporting Information/S4_File_Forest_Data.docx.docx]

**Corresponding to the specific data in the forest plot**

**GMFM-score**

|  | GMFM-score | Exr | | | Con | | |
| --- | --- | --- | --- | --- | --- | --- | --- |
|  |  | M | SD | n | M | SD | n |
|  | ZHANG Jianfeng et al. 2023 | 87.72 | 7.81 | 100 | 73.26 | 6.38 | 100 |
| 总 | Yang Yali et al. 2023 | 83.83 | 4.67 | 40 | 74.1 | 4.41 | 40 |
| D | Yang Yali et al. 2023 | 31.28 | 2.31 | 40 | 27.68 | 1.98 | 40 |
| E | Yang Yali et al. 2023 | 52.55 | 3.94 | 40 | 46.43 | 3.75 | 40 |
| D | Kvedaravičienė, K et al. 2020 | 90.5 | 4.3 | 10 | 89.4 | 4 | 10 |
| E | Kvedaravičienė, K et al. 2021 | 81.9 | 5.9 | 10 | 79.8 | 5.8 | 10 |
| D | Lee, NY et al. 2021 | 82.57 | 31.07 | 7 | 69.57 | 39.86 | 7 |
| E | Lee, NY et al. 2022 | 79.28 | 31.11 | 7 | 64.14 | 37.83 | 7 |
| 总 | Lee, NY et al. 2023 | 90.71 | 15.14 | 7 | 82.57 | 21.67 | 7 |
| D | Guangjin, L et al. 2022 | 36.28 | 1.99 | 18 | 35.88 | 1.82 | 16 |
| E | Guangjin, L et al. 2022 | 53.94 | 6.98 | 18 | 51.2 | 6.64 | 16 |
| D | Szturm, T et al. 2022 | 29.5 | 4.2 | 10 | 28 | 4.2 | 10 |
| E | Szturm, T et al. 2023 | 34.7 | 7.4 | 10 | 35.3 | 6.6 | 10 |

**PBS**

| **PBS** | Exr | | | Con | | |
| --- | --- | --- | --- | --- | --- | --- |
|  | M | SD | N | M | SD | N |
| Mahmoud, A et al. 2023 | 41 | 8 | 17 | 43.58 | 7.36 | 17 |
| Kvedaravičienė, K et al. 2020 | 34.68 | 1.1 | 10 | 32.2 | 1.3 | 10 |
| Guangjin, L et al. 2022 | 43.06 | 43.06 | 18 | 40.75 | 4.14 | 16 |
| Uysal, I et al. 2024 | 45.46 | 5.77 | 15 | 44.06 | 4.99 | 15 |
| Szturm, T et al. 2022 | 35.6 | 4.4 | 10 | 30.6 | 3.5 | 10 |
| Kamran, S et al. 2023 | 41 | 4 | 26 | 37 | 3 | 16 |

**Walking Ability**

| **Walking Ability** | **Exr** | | | **Con** | | |
| --- | --- | --- | --- | --- | --- | --- |
|  | M | SD | N | M | SD | N |
| ZHANG Jianfeng et al. 2023 | 66.72 | 8.32 | 100 | 62.68 | 7.15 | 100 |
| Yang Yali et al. 2023 | 52.2 | 20.4 | 40 | 41.4 | 16.2 | 40 |
| LUO Shui-ming 2021 et al. 2023(China) | 66 | 4.2 | 29 | 62.4 | 2.4 | 29 |
| Kvedaravičienė, K et al. 2020 | 77.23 | 1.02 | 10 | 74.83 | 1.1 | 10 |
| Guangjin, L et al. 2022 | 10.44 | 2.83 | 16 | 10.81 | 2.95 | 16 |
| Uysal, I et al. 2024 | 8.84 | 2.44 | 15 | 8.14 | 2.37 | 15 |
| Mohammed Omar Abuzaid, S et al. 2024 | 21.15 | 3.11 | 6 | 16.9 | 5.44 | 6 |

**BBS-static balance**

|  | BBS-static balance | Exr | | | Con | | |
| --- | --- | --- | --- | --- | --- | --- | --- |
|  |  | M | SD | N | M | SD | N |
|  | ZHANG Jianfeng et al. 2023 | 12.94 | 2.15 | 100 | 10.26 | 2.01 | 100 |
| Open | Mahmoud, A et al. 2023 | 75.58 | 11.8 | 17 | 78.76 | 7.94 | 17 |
| Closed | Mahmoud, A et al. 2024 | 68.76 | 13.45 | 17 | 75.88 | 9.7 | 17 |
| Open | Uysal, I et al. 2024 | 7.16 | 8.3 | 15 | 6.1 | 5.13 | 15 |
| Closed | Uysal, I et al. 2025 | 5.38 | 7.1 | 15 | 3.8 | 3.91 | 15 |
| OPen | Szturm, T et al. 2022 | 39.3 | 5.7 | 10 | 44.6 | 5.5 | 10 |
| Closed | Szturm, T et al. 2023 | 48.3 | 5.4 | 10 | 42.7 | 4.2 | 10 |

**Dynamic balance**

| BBS-dynamic balance | Exr | | | Con | | |
| --- | --- | --- | --- | --- | --- | --- |
|  | M | SD | N | M | SD | N |
| ZHANG Jianfeng et al. 2023 | 14.61 | 2.32 | 100 | 10.57 | 2.26 | 100 |
| Yang Yali et al. 2023 | 42.8 | 4.26 | 40 | 37.67 | 2.95 | 40 |
| LUO Shui-ming 2021 et al. 2023(China) | 48.38 | 3.98 | 29 | 45 | 4.41 | 29 |
| Guangjin, L et al. 2022 | 10.56 | 2.73 | 16 | 10.06 | 2.52 | 16 |
| Uysal, I et al. 2024 | 7.75 | 2.02 | 15 | 8.2 | 2.16 | 15 |
| Mohammed Omar Abuzaid, S et al. 2024 | 11.15 | 3.11 | 6 | 16.3 | 5.4 | 6 |

**Literature Selection Process**

| **Stage** | **Description** | **Number** | **Notes** |
| --- | --- | --- | --- |
| Identification | Records identified from Databases | 357 |  |
| Identification | Records identified from Registers |  |  |
| Identification | Records removed before screening (Duplicate, Ineligible, Other reasons) | 112 (Duplicate) | Duplicate records removed, |
| Screening | Records screened | 315 | Records excluded (n = 273)  --Review (n = 204)  --Not relevant to topic (n = 69) |
| Screening | Reports sought for retrieval | 42 | Full-texts articles excluded (n = 29)  --Not RCT (n =6)  ---Not -DDT (n = 11)  --Not CP patients (n = 9)  --Full text could not be found (n = 3) |
| Screening | Reports assessed for eligibility | 13 |  |
| Screening | Reports excluded (Reviews, commentaries, animal experiments) | 2 (Reviews, commentaries, animal experiments) | Inconsistent interventions or treatments, Insufficient data, Outcome measures inconsistent |
| Included | Studies included in review | 11 |  |
